# Experimental Validation of Two Types of Force Actuators: A Performance Comparison

**DOI:** 10.3390/s24123950

**Published:** 2024-06-18

**Authors:** Xishan Jiang, Ning Wang, Jing Zheng, Jie Pan

**Affiliations:** 1 Department of Instrument Science and Engineering, Zhejiang University, Hangzhou 310027, China; jxishan@zju.edu.cn (X.J.); zjuningwang@163.com (N.W.); 10915008@zju.edu.cn (J.Z.); 2 Department of Mechanical Engineering, University of Western Australia, Crawley, WA 6009, Australia

**Keywords:** piezoelectric stack actuator, transmitted force, power flow, frame-type actuator

## Abstract

This paper experimentally investigates the performance of piezoelectric force actuators. Using the same encapsulated piezoelectric stack, an inertial-type actuator and a frame-type actuator are constructed for performance comparison. The experimental results are also used to validate the recently established actuator models, whilst the mechanical and piezoelectrical parameters of the models are experimentally identified. The performance of the actuators is described by the transmitted force(s) and input power flow from the actuators to the base structure with reference to the same electrical input voltage to the stack. The validation is deemed successful due to the strong agreement observed between the measured and predicted actuator performances. Additionally, it is discovered that the frame-type actuator has the capacity to produce significantly higher transmitted forces and input power flow to the base structure compared to the inertial-type actuator. The mechanism underlying the performance disparity between these two types of actuators is also examined. This paper clarifies the mechanism, shedding light on the design and optimization of piezoelectric actuators.

## 1. Introduction

Piezoelectric actuators belong to a category of driving devices that exploit the inverse piezoelectric effect present in piezoelectric materials. This phenomenon enables the conversion of electrical energy into mechanical energy, which can then be utilized to induce displacements or apply forces [1]. Due to their lightweight nature, high resolution, accuracy, and ease of control, they have found extensive use in fast steering mirrors [2,3], nanopositioning stages [4,5] and structural dynamics applications [6,7]. Over the past few decades, the design principles governing piezoelectric actuators have undergone significant evolution, leading to the development of various types of actuators. Among these, piezoelectric stack actuators possess several advantageous characteristics, including rapid response times, low power consumption, and the ability to generate outputs with enhanced precision in terms of the displacement and greater force generation capabilities [8,9,10].

While piezoelectric stack actuators are capable of directly actuating structures, auxiliary preloading devices are often necessary to ensure effective output of the driving force. The inertial mass has commonly been employed in the development of inertial-type actuators to achieve larger output forces [11,12]. Tianyue Pan et al. contributed to the development of an inertial piezoelectric actuator and investigated its dynamic characteristics [13]. Seung-Bok Choi et al. designed a novel inertial-type actuator incorporating a piezoelectric stack actuator and explored the relationship between the actuating force and the applied voltage [14]. However, to ensure stable installation, the actuating force direction of these inertial-type actuators was typically constrained to the direction of gravity, thus limiting their applications. Furthermore, the nonlinear hysteresis of piezoelectric materials was often not thoroughly examined when evaluating the steady-state performance of the actuators.

Recently, authors proposed a new frame-type actuator wherein a piezoelectric stack is integrated into a frame structure. This configuration was devised to eliminate the need for traditional “earth-connected” support and enable the transmission of force in any direction [15]. When comparing the performance of the frame-type actuator with that of the inertial-type actuator, it was noted that the frame-type actuator exhibited an advantage in generating larger forces. Motivated by this observation, the present study initially focuses on experimentally comparing the transmitted forces from the actuators to the base structure, thereby validating the previously established models of the aforementioned actuators. Since the capacity of a force actuator to excite the base structure depends not only on the transmitted force but also on the response of the base structure, it is necessary to consider the interaction between the actuator and the elastic base structure. The concept of power flow through the interface between the actuator and the base structure emerges as a valuable tool to describe this coupling effect. It encompasses both the force and velocity responses of the structure, providing a comprehensive representation of energy transmission into the structure. Essentially, it quantifies the rate at which vibrational energy is transmitted into the base structure, offering a scientific framework for analyzing vibration problems in terms of energy transmission [16,17].

To thoroughly assess the actuator’s performance, both the force transmitted to the base structure and the power flow between the actuator and the base structure are examined. By considering the power flow and its components, a more comprehensive evaluation of the actuator’s ability to energize and interact with the base structure is achieved.

This paper is structured as follows. Section 2 reviews the analytical models of the inertial-type and frame-type actuators, along with introducing a composite method of parameter identification for the dynamics model of the actuators. Section 3 summarizes the experimental methods employed. In Section 4, the measured performance of the two actuators is investigated and compared. Section 5 succinctly outlines the experimental results and the performance comparison between the two types of actuators. Additionally, potential applications of the frame-type actuator are briefly discussed within the same section. This experimental work not only validates the theoretical predictions but also enhances understanding of the important factors influencing the performance of two type of actuators, which can be useful for future design and application of piezoelectric actuators. 

## 2. Modeling of the Actuators

**Figure 1:**
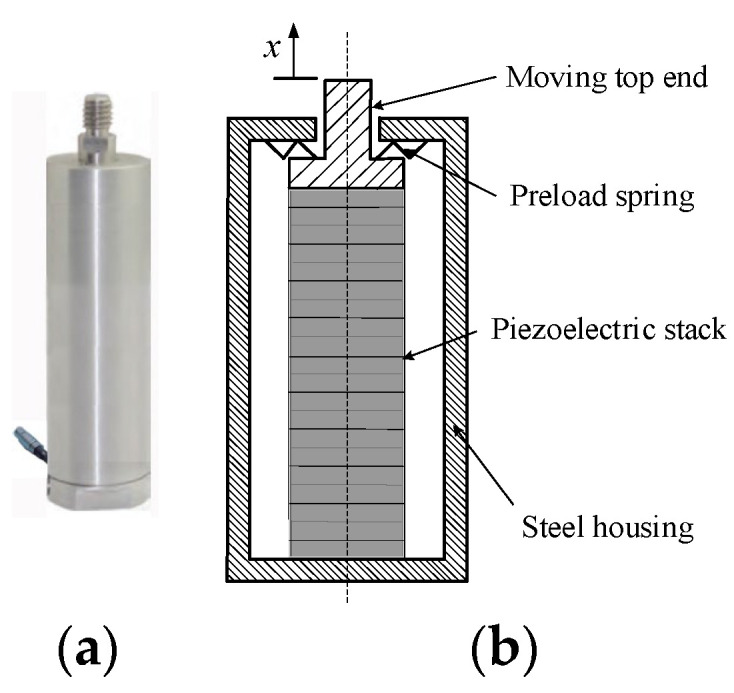
Configuration of the encased piezoelectric stack actuator (EPSA): (**a**) photograph; and (**b**) schematic configuration.

Piezoelectric ceramics are frequently stacked in layers to enhance the displacement and force. This configuration effectively links each piezoelectric layer mechanically in series and electrically in parallel [18]. While this arrangement offers high stiffness in the axial direction, it is susceptible to lateral forces that could potentially harm the actuator. Consequently, to enhance the actuator’s lateral strength, a steel housing with a preload spring is commonly employed to shield the piezoelectric ceramics, as depicted in Figure 1. The application of preload force significantly reduces the lateral movement of the piezoelectric ceramics.

This initial condition needs to be considered in the modeling process. The output displacement of the actuator was modeled by the Bouc–Wen model [18],

(1) $$\left\{\begin{array}{l} y(t)=d_{p}V(t)-h(t)\\ \dot{h}=\alpha \dot{V}-\beta |\dot{V}|h|h{|}^{n-1}-\gamma \dot{V}|h{|}^{n} \end{array}\right.$$

where the first equation relates the driving signal V(t) to the actuator deformation y(t) and with hysteresis displacement h(t). $d_{p}$ is the piezoelectric coefficient, which characterizes the electromechanical transfer coefficient. Parameters α, β, and γ are the constants affected by the shape of the hysteresis curve. For a PZT actuator, *n* = 1 is generally used [19,20].

The deformation y(t) could then be related to the dynamic model, where the displacement of the piezoelectric material could be converted into actuating forces. As mentioned before, to achieve a better actuating performance, new designs of the piezo actuator, including an inertial-type actuator and a frame-type actuator, have been proposed. Two dynamic models describing the mechanical behavior of the two actuators have also been developed accordingly. In the following sections, these two models will be briefly reviewed. Readers interested in the details are directed to references [15,21].

### 2.1. Inertial-Type Actuator 

**Figure 2:**
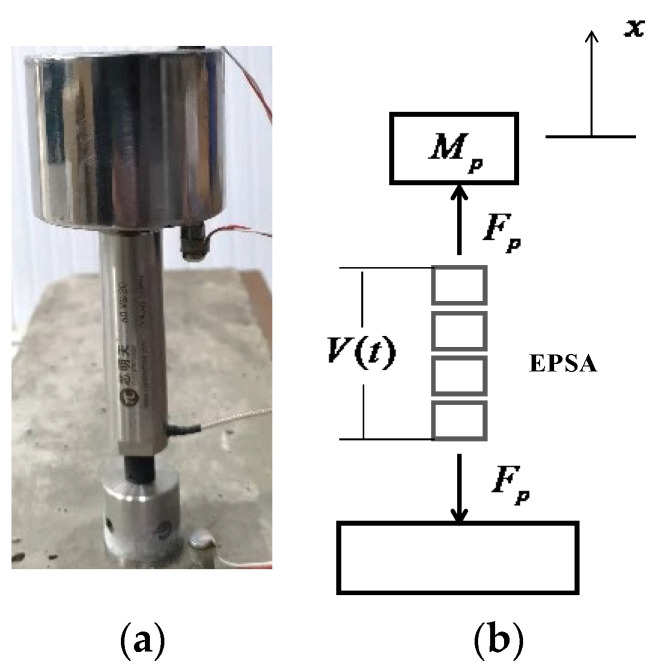
Inertial-type actuator: (**a**) photograph; and (**b**) lumped-element model.

An inertial-type actuator is developed by fixing an inertial mass to the top end of the EPSA, as shown in Figure 2. $F_{p}$ is the PZT electric force from EPSA in Figure 2b.

Previous work [21] described the dynamics of the inertial-type actuator as follows:

(2) $$\left\{\begin{array}{l} \left(M_{p}+m_{p})\ddot{x}+b_{p}\dot{x}+k_{p}x=k_{p}(d_{p}V-h\right)\\ \dot{h}=\alpha \dot{V}-\beta \left|\dot{V}\right|h-\gamma \dot{V}\left|h\right| \end{array}\right.$$

where x(t) and $M_{p}$ are the displacement and mass of the top mass. $m_{p}$, $b_{p}$ and $k_{p}$ are, respectively, the mass, damping, and stiffness of the EPSA. The actuator is excited by the deformation of the piezo material y(t), as described in Equation (1), and is rewritten as $\left(d_{p}V-h\right)$. The transmitted force generated by the actuator to the solid base could be calculated by:

(3) $$F_{T}(t)=b_{p}\dot{x}+k_{p}x-k_{p}(d_{p}V-h)$$

### 2.2. Frame-Type Actuator

Inertial-type actuators have a simple structure and are widely used. A challenge emerges with inertial-type actuators when utilized for low-frequency actuation from directions other than vertical. The gravitational moment generated by the top mass can result in the static instability of the actuator. The frame-type actuator is engineered to circumvent the need for “earth-connected” support, facilitating easy attachment to steel structures from any direction. In addition, this type of actuator can generate a driving force of hundreds or even thousands of Newtons.

**Figure 3:**
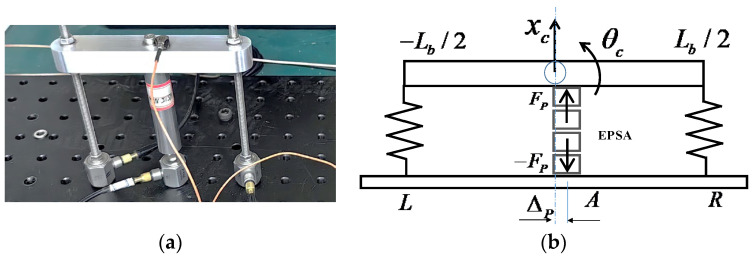
Frame-type piezoelectric actuator: (**a**) photograph; and (**b**) lumped-element model.

Figure 3 illustrates a photograph of a frame-type actuator alongside the lumped element model of the actuator. 

The model of the frame-type actuator is established as follows. The EPSA is driven by a time-dependent voltage V(t) applied to it. The top beam is approximated as a rigid body with mass $M_{b}$ and length $L_{b}$. The motion of it is described by the displacement $x_{c}$ of the mass center and angular displacement $\theta_{c}$ about the center. The reason for the twisting/bending load is the existence of the unbalanced loading described by the distance of the EPSA from the center of the beam $\Delta_{P}$. In practical applications, it should be minimized as much as possible. The actuator is excited by PZT electric force $F_{p}$. The dynamics of the actuator, as investigated in prior research [15], are revisited through the following equations, where a rigid base structure is assumed.

(4) $$\begin{array}{l} M_{b}{\ddot{x}}_{c}+b_{p}({\dot{x}}_{c}+\Delta_{P}{\dot{\theta }}_{c})+k_{p}(x_{c}+\Delta_{P}\theta_{c})+b_{f}({\dot{x}}_{c}+\frac{L}{2}{\dot{\theta }}_{c})\\ +b_{f}({\dot{x}}_{c}-\frac{L}{2}{\dot{\theta }}_{c})+k_{f}(x_{c}+\frac{L}{2}\theta_{c})+k_{f}(x_{c}-\frac{L}{2}\theta_{c})=k_{p}(d_{p}V-h) \end{array}$$

(5) $$\begin{array}{l} I_{b}{\ddot{\theta }}_{c}+b_{p}({\dot{x}}_{c}+\Delta_{P}{\dot{\theta }}_{c})\Delta_{P}+k_{p}(x_{c}+\Delta_{P}\theta_{c})\Delta_{P}+b_{f}({\dot{x}}_{c}+\frac{L}{2}{\dot{\theta }}_{c})\frac{L}{2}\\ -b_{f}({\dot{x}}_{c}-\frac{L}{2}{\dot{\theta }}_{c})\frac{L}{2}+k_{f}(x_{c}+\frac{L}{2}\theta_{c})\frac{L}{2}-k_{f}(x_{c}-\frac{L}{2}\theta_{c})\frac{L}{2}=k_{p}(d_{p}V-h)\Delta_{P} \end{array}$$

where $k_{p}$, $k_{f}$, $b_{p}$ and $b_{f}$ are constants and represent, respectively, the stiffness and damping of the EPSA and the legs of the frame. $I_{b}$ is the mass moment inertia of the top beam. Thus, the forces transmitted through the EPSA and the right and left legs of the frame to the rigid base can be described as:

(6) $$F_{L}(t)=k_{f}(x_{c}-\frac{L}{2}\theta_{c})+b_{f}({\dot{x}}_{c}-\frac{L}{2}{\dot{\theta }}_{c}),$$

(7) $$F_{A}(t)=b_{p}({\dot{x}}_{c}+\Delta_{P}{\dot{\theta }}_{c})+k_{p}(x_{c}+\Delta_{P}\theta_{c})-k_{p}(d_{p}V-h),\;\mathrm{and}$$

(8) $$F_{R}(t)=k_{f}(x_{c}+\frac{L}{2}\theta_{c})+b_{f}({\dot{x}}_{c}+\frac{L}{2}{\dot{\theta }}_{c}).$$

### 2.3. Parameter Identification

To validate the dynamic models based on the Bouc–Wen model, the piezoelectric coefficient $d_{p}$, the hysteresis parameters α, β, γ and the dynamic parameters $b_{p}$, $k_{p}$ in Equations (1)–(8) need be identified.

Following the methods described in reference [22], two driving voltage signals $u_{1}(t)$ and $u_{2}(t)$ with the same cycles *T* are used to excite the actuator, where $u_{2}(t)=u_{1}(t)+\Delta u$ and Δu is constant. The corresponding output displacements in Equation (1) can be expressed as follows:

(9) $$y_{1}(t)=d_{p}u_{1}(t)-h(t)$$

(10) $$y_{2}(t)=d_{p}u_{2}(t)-h(t)$$

When *M* sets of sampling points ([$u_{1},y_{1}$], [$u_{2},y_{2}$], …, [$u_{M},y_{M}$]) are used, the piezoelectric coefficient $d_{p}$ can be obtained based on Equation (4):

(11) $$d_{p}=\frac{\sum_{i=1}^{n}\left(y_{1}{\;}_{i}-y_{2}{\;}_{i}\right)}{M\Delta u}$$

Using the Particle Swarm Optimization (PSO) method [23], the hysteresis parameters α, β and γ of the EPSA in Equation (1) are identified. It should be pointed out that the hysteresis parameters α, β and γ in Equation (1) do not have physical meanings. To ensure the physical and mathematical consistency of the Bouc–Wen model, some of the constraints need to be met [24], as listed in Table 1.

The dynamic parameters $b_{p}$ and $k_{p}$ in Equation (2) are obtained by Experimental Modal Analysis (EMA) [25]. The impact hammer that acts at the center of the top surface of the mass is used to generate an input force, and an accelerator located close to the force is used to measure the response. Then, the dynamic parameters are calculated by the frequency response function (FRF) based on the measured input force and acceleration response.

All the parameters used for modeling the actuator performance are listed in Table 2.

## 3. Experimental Methods

This section describes the experimental details for testing the performances of the two actuators.

The experimental configurations are crafted to evaluate the efficacy of the two actuators, specifically targeting the measurement of the transmitted force(s) and power flow to the base structures from each actuator. 

The measurements are performed on two distinct base structures (a rigid base structure and an elastic beam structure) for each actuator. An EPSA made of a piezoelectric stack (COREMORROW, PSt150/10/60 VS15, material: PZT-5, dimensions: 7 mm × 7 mm × 20 mm) (CoreMorrow, Harbin, China) in a steel housing is used.

### 3.1. Transmitted Force Measurement

**Figure 4:**
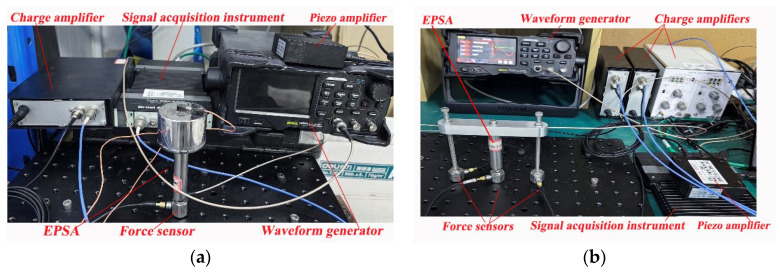
Test platform for the transmitted forces: (**a**) experimental setup for testing the inertial-type actuator; and (**b**) experimental setup for testing the frame-type actuator.

To measure the transmitted force, the actuators are fixed to the rigid base and activated by the waveform generator (RIGOL, DG4000, RIGOL Technologies, Beijing, China) and the piezo amplifier (COREMORROW, E53.A, CoreMorrow, Harbin, China), as shown in Figure 4. The signal acquisition instrument (B&K, 4517-002, Brüel & Kjær, Nærum, Denmark) is used for collecting the measured force signals from the force sensors. For the inertial-type actuator, one force sensor (TRCG, P-F02, sensitivity: 4pC/N, Econ, Hanghzou, China) and one charge amplifier (TIRA, Model 4578A, Tira, Mumbai, India) are used to measure the transmitted force, while for the frame-type actuator, three force sensors are used to determine the transmitted forces at the feet of the EPSA and the two frame legs.

### 3.2. Power Flow Measurement

Power refers to the work performed per unit of time, and the instantaneous power input to the structure is defined as [26]:

(12) $$P_{i}=F_{i}V_{i}$$

where $F_{i}$ and $V_{i}$ are the instantaneous values of the force and velocity at the driving point. The time-averaged vibrational flow of power is usually used when analyzing a vibrating structure. When the force and velocity are harmonic, the time-averaged vibrational flow can be written as:

(13) $$P=\frac{1}{T}\int_{0}^{T}F_{i}V_{i}dt$$

where $F_{i}=\tilde{F}e^{i\omega t}$ and $V_{i}=\tilde{V}e^{i\omega t}$. Then, the vibrational power is given by

(14) P=12ReF˜∗V˜=12ReF˜V˜∗

When the acceleration on a structure is used instead of the velocity, Equation (14) can be rewritten as:

(15) P=−12ωImF˜a˜∗

where ${\tilde{a}}^{*}$ is the complex conjugate of $\tilde{a}$ and $\tilde{a}=i\omega \tilde{V}$. 

**Figure 5:**
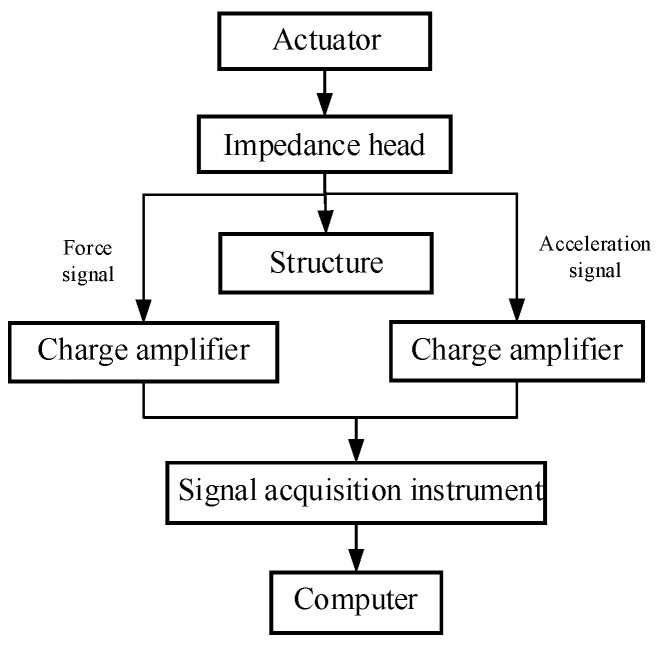
The flow chart of the measurement of the power flow.

The calculation of the power flow necessitates the concurrent measurement of the force and acceleration at a single location, which can pose practical challenges due to constraints in force sensor fixation [27]. In this study, by inserting an impedance head between the actuator and the structure, it is possible to simultaneously capture both the force signal and the acceleration signal from the same location and direction. Figure 5 shows the flow chart of the measurement of the power flow. 

**Figure 6:**
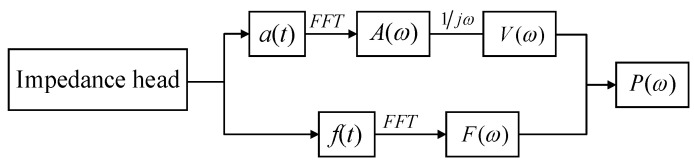
The flow chart of the calculation of the power flow.

The post-processing of the measurement data can be performed in MATLAB, which is presented in Figure 6. Firstly, the impedance sensor obtains the force and acceleration signals. Secondly, based on the Fourier transform property, the acceleration signal a(ω) is converted into a velocity signal V(ω). Then, the power flow P(ω) can be obtained in Equation (15).

**Figure 7:**
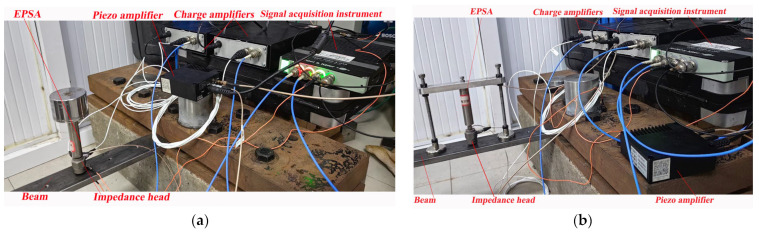
Test platform for the power flow: (**a**) experimental setup for testing the inertial-type actuator; and (**b**) experimental setup for testing the frame-type actuator.

To assess the power flow from the two actuators to the base structure, a clamped-clamped steel beam serves as the foundation. As depicted in Figure 7, the actuators are securely mounted onto the beam. An impedance head (TIRA Model 5860B) along with two charge amplifiers (TIRA, Model 4578A) are utilized to measure the input power flow to the beam at the interface between the EPSA and the beam. To evaluate the power flow at the feet of the two frame legs of the frame-type actuator, we position the same impedance head at the junction of each leg and the base beam.

## 4. Result and Discussion

### 4.1. Transmitted Forces

**Figure 8:**
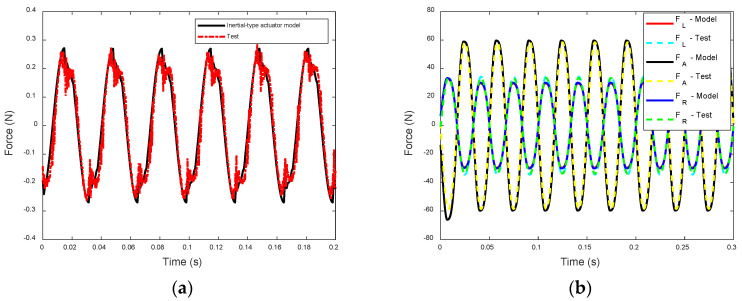
The measured and predicted transmitted forces of: (**a**) the inertial-type actuator time domain; and (**b**) the frame-type actuator.

To measure the transmitted forces of the two actuators to the rigid base structure, a sinusoidal voltage with a magnitude of 48 V and a frequency of 30 Hz is applied as input to the EPSA, and the forces are then measured using force sensors. A comparison between the measured and predicted forces is illustrated in Figure 8, where the predicted forces are calculated using the dynamic models and system parameters outlined in previous sections. It is evident from the comparison that there is a favorable agreement between the test and prediction. The overall error between the experimental results and the analytical and numerical results is less than 1 dB at 30 Hz.

Upon comparing the transmitted forces of the two actuators, it becomes apparent that the frame-type actuator is capable of generating significantly higher transmitted forces. In fact, it exceeds those of the inertial-type actuator by more than two orders of magnitude.

**Figure 9:**
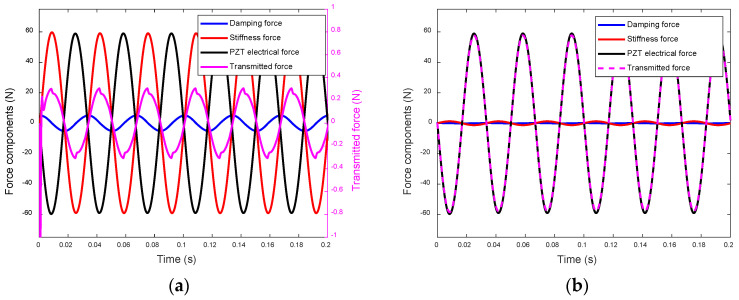
The force components of: (**a**) the inertial-type actuator (*y*-axis of transmitted force on the right-hand side); and (**b**) the frame-type actuator.

To elucidate the magnitude difference in the transmitted forces of the actuators, we analyze the components of the transmitted force at the bottom of the EPSA. As depicted in Equations (3) and (7), each transmitted force comprises damping, stiffness, and PZT electrical force components. Figure 9 presents the simulated component forces and transmitted force of the inertial-type actuator, as well as those at the foot of the EPSA of the frame-type actuator.

Given the same input voltage, the PZT electrical force components in the transmitted forces of the two actuators are identical. However, the stiffness force component in $F_{T}(t)$ (the transmitted force of the inertial-type actuator) is much larger than that in $F_{A}(t)$ (the transmitted force of the frame-type actuator). The damping force components are generally small and have less influence on the transmitted forces.

In the case of the inertia-type actuator, as depicted in Figure 9a, the stiffness force component exhibits an opposite phase to the PZT electrical force component, albeit with a slightly larger magnitude. These two components undergo a destructive superposition, resulting in the reduced magnitude of the transmitted force. Conversely, for the frame-type actuator, the stiffness force component is considerably smaller than the PZT electrical force component. Consequently, the electrical component remains largely unaffected after the superposition, leading to a much larger transmitted force.

**Figure 10:**
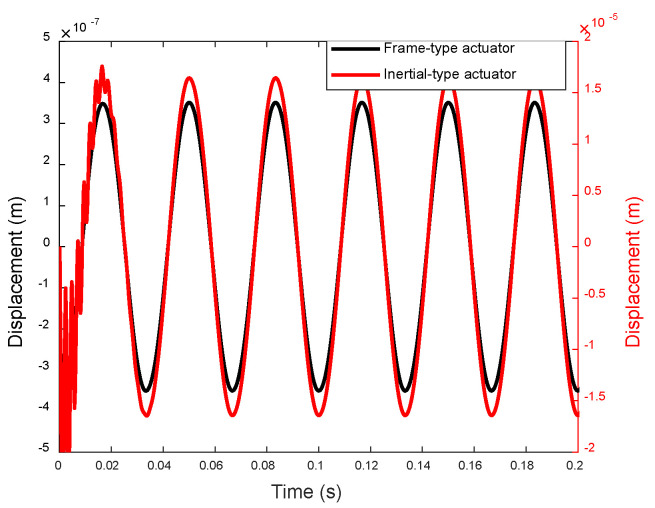
Displacement of two types of actuators.

The notable differences in the magnitudes of the stiffness force components stem from the distinct configurations of the two types of force actuators and the resulting discrepancies in the displacements atop the actuators. As illustrated in Figure 10, the simulated displacement of the two actuators indicates that the displacement at the top beam of the frame-type actuator is two orders of magnitude smaller than that at the top mass of the inertia-type actuator. Piezoelectric ceramics generate the same electrical displacement and electromagnetic force *F_p_* under the same voltage excitation in the two systems. As shown in Figure 2b, the electromagnetic force Fp acts on the inertial mass and rigid base, while in the frame-type actuator (Figure 3b), the upper part of the electromagnetic force Fp is limited by the frame and is transmitted to the base through the two supporting feet. The two systems have different structures and boundary conditions, resulting in different system displacement responses.

**Figure 11:**
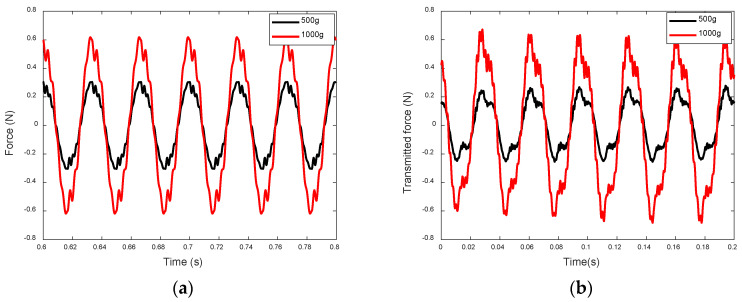
Transmitted forces from the inertial-type actuator with different top mass (input voltage: 48 V): (**a**) predicted data; and (**b**) measured data.

When actuated, the top mass of the inertia-type actuator vibrates at the free end and typically exhibits a large amplitude. With low-frequency excitation and a fixed stiffness of the EPSA, elevating the top mass shifts the actuator’s natural frequency closer to the driving frequency, thereby enhancing the displacement response. Consequently, to achieve a larger transmitted force through the EPSA, it becomes necessary to increase the mass of the inertia-type actuator, as demonstrated by the transmitted forces from inertia-type actuators with varying top masses shown in Figure 11. On the other hand, for the frame-type actuator, the vibration of the frame is structurally confined since the frame is anchored to the base by its two legs. This limitation on the stiffness force component ensures a larger total transmitted force at the bottom of the EPSA. Moreover, the movement of the top beam and the substantial stiffness constant of the frame’s legs also enable significantly larger transmitted forces at the feet of the frame’s legs. Therefore, compared with the inertia-type actuator, the frame-type actuator can achieve greater driving force capability with less weight.

It is also noticed in Figure 8b that the force $F_{A}(t)$ from the EPSA has a larger magnitude than the other two forces and it has an opposite phase to $F_{L}(t)$ and $F_{R}(t)$. This feature of the actuator is important when the input power flow of the actuator is calculated.

**Figure 12:**
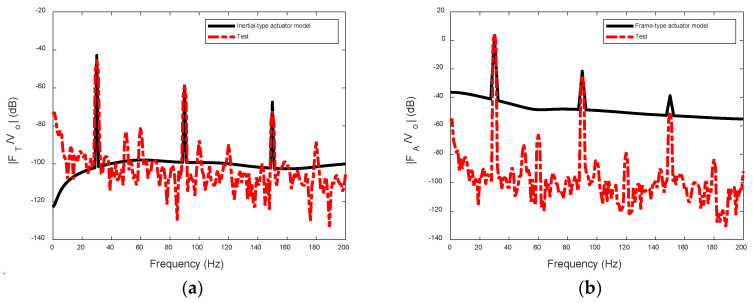
Frequency responses of the transmitted forces of the two actuators: (**a**) inertial-type actuator; and (**b**) $F_{A}(t)$ of frame-type actuator.

Figure 12 displays the frequency responses of the transmitted forces of the two actuators. These force spectra are typically characterized by the force components at the driving frequency and its odd harmonics. Additionally, it is observed that the response at the driving frequency of the frame-type actuator is significantly amplified, effectively mitigating interferences caused by even components and 50 Hz noises. The discrepancy between the test and predicted results comes from the influence of the 50 Hz driving current and its harmonic components. However, this background noise in the test result is less than the signal at 30 Hz and its odd harmonics by more than 15 dB, and it does not affect the conclusion.

### 4.2. Power Flow

Drawing on our understanding of the actuator’s excitation mechanism, we delve into the investigation of the steady-state interaction between the actuator and the beam. Velocity and transmitted force measurements taken at the attachment locations enable the determination of the power flow between the force actuator and the structure.

**Figure 13:**
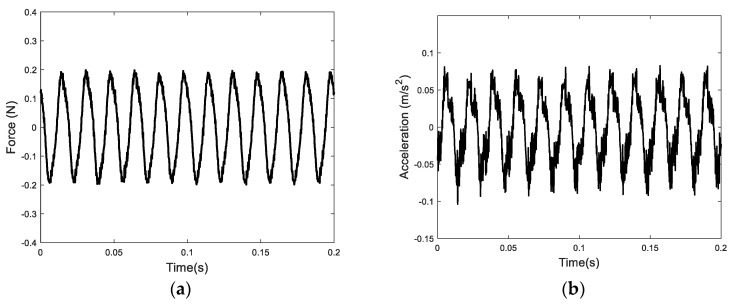
The measured force and acceleration of the impedance head under the inertial-type actuator: (**a**) time histories of the force; and (**b**) time histories of the acceleration.

**Figure 14:**
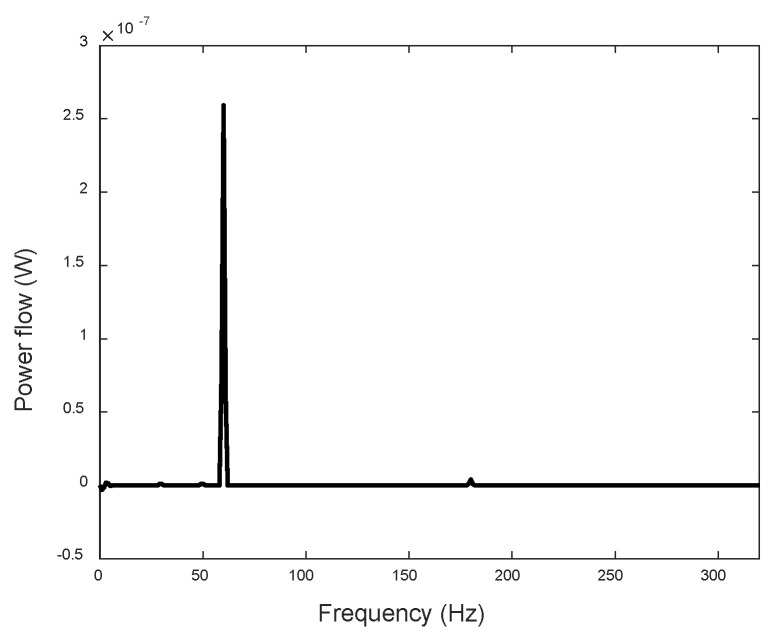
Input power flow from the inertial-type actuator to the beam.

Each actuator’s EPSA is positioned 150 mm away from one end of the beam, as depicted in Figure 7. The beam, with two clamped ends, has dimensions of 730 mm in length, 50 mm in width, and 5 mm in thickness. Employing a sinusoidal excitation voltage at 60 Hz and 12 V, the force and acceleration signals at the attachment location between the inertia-type actuator and the beam are captured, as shown in Figure 13. Subsequently, the input power flow from the inertia-type actuator to the beam is calculated and depicted in Figure 14. Notably, it is evident that the primary power flow occurs at 60 Hz and 180 Hz.

**Figure 15:**
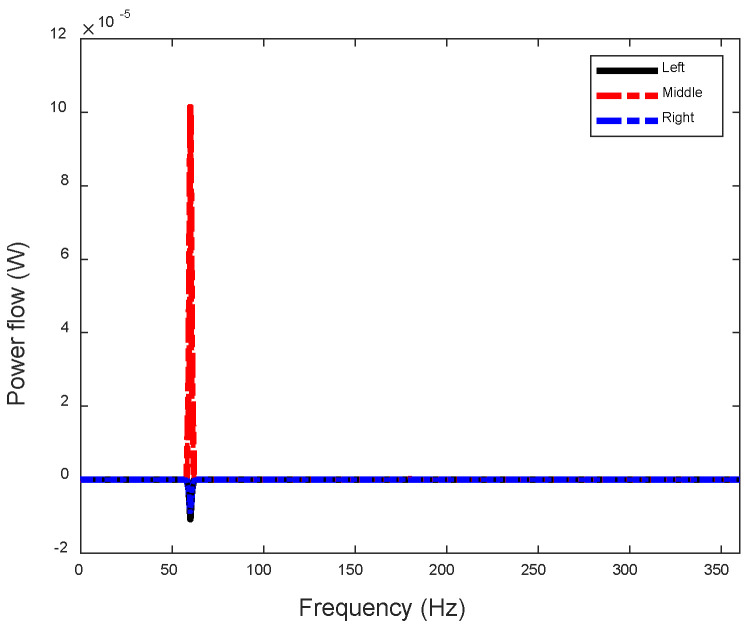
Input power flow from the frame-type actuator to the beam.

Likewise, the power flow at the three attachment locations of the frame-type actuator can be derived, as illustrated in Figure 15. The power flow components at the frame legs exhibit an opposite sign from those through the EPSA (middle position), indicating that the actuator injects power into the beam through the EPSA, but some of it returns to the actuator through the two frame legs.

**Figure 16:**
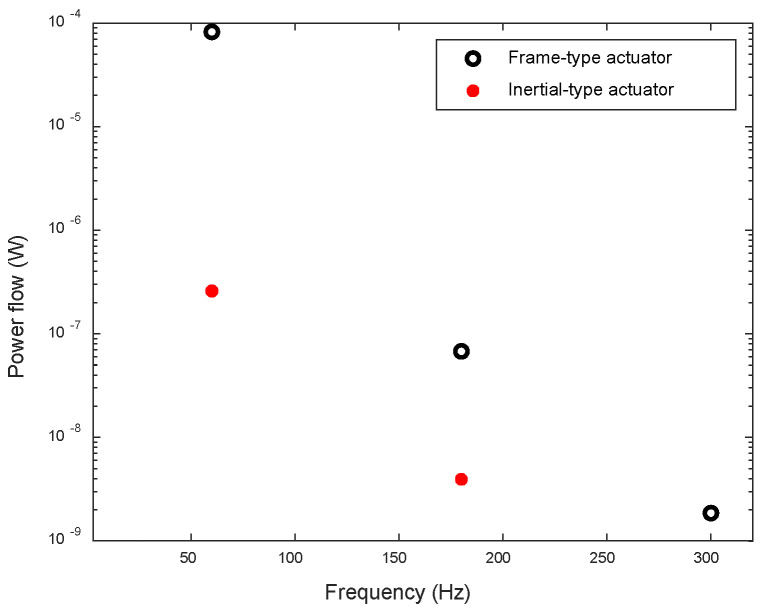
Total input power flow from the actuators to the beam.

The total power flow of the frame-type actuator can be determined by summing up the power flow transmitted through the three attachment points. Figure 16 illustrates the net power flows into the beam from the actuators. At the driving frequency, the total input power flow of the frame-type actuator is approximately 300 times higher than that of the inertial actuator.

## 5. Conclusions

This paper undertakes an experimental investigation into two types of force actuators, with the aim of validating previously developed dynamic models based on the Bouc–Wen model and the identified parameters of these models. The experimental findings confirm that the transmitted forces predicted by the dynamic models closely match the measured data.

Moreover, two distinct test platforms are established to evaluate the performance of the two types of force actuators. The measurements of transmitted forces demonstrate that the frame-type actuator is capable of generating significantly larger transmitted forces compared to the inertial one.

The ability of the actuator to stimulate the structure is heavily influenced by the interaction between the force actuator and the structure. By measuring the input power flow at the attachment locations through an impedance head, the steady-state interaction between the actuator and the beam is investigated. Both the experimental and calculated results indicate that the frame-type actuator can generate a significantly greater power flow to the beam compared to the inertial-type one.

In conclusion, this experimental study provides a comprehensive assessment of two types of force actuators, emphasizing the advantages of the frame-type actuator in terms of the force generation and power flow input into the structure. The research not only validates the theoretical predictions but also enhances our understanding of the factors influencing the performance of these actuators, thereby guiding future advancements in actuator technology. Future work will focus on discussing methods to suppress the nonlinear effects of frame-type actuators and their utilization in the active control of structural vibration.

## Figures and Tables

**Table 1:** Constraints of the parameters α, β and γ in the Bouc–Wen model.

Parameters	α	β	γ
Constraints	α>0	β> 0	β + γ > 0 and β − γ ≥ 0

**Table 2:** Parameters identified for modeling the actuator performance.

Parameters	Value
$M_{p}$	0.5 kg
$m_{p}$	0.004 kg
$b_{p}$	163.65 Ns/m
$k_{p}$	$3.61\times 10^{6}$ N/m
$d_{p}$	$2.19\times 10^{-7}$ m/V
α	$3.58\times 10^{-8}$
β	0.0214
γ	0.0037
$L_{b}$	0.12 m
*W* (width of the top-beam)	0.02 m
*T* (thickness of the top-beam)	0.01 m
$I_{b}=M_{b}L_{b}^{2}/12$	$7.776\times 10^{-5}\;{\mathrm{kgm}}^{2}$
$M_{b}=2700\times L_{b}\times W\times T$	0.0648 kg
$b_{f}$	160 Ns/m
$k_{f}$	$8.2291\times 10^{7}N/m$

## Data Availability

The data presented in this study are available on request from the corresponding author. The data are not publicly available due to a confidentiality agreement for the project.
